# High-resolution profiling of the gut microbiome reveals the extent of *Clostridium difficile* burden

**DOI:** 10.1038/s41522-017-0043-0

**Published:** 2017-12-05

**Authors:** Ninalynn Daquigan, Anna Maria Seekatz, K. Leigh Greathouse, Vincent B. Young, James Robert White

**Affiliations:** 1 Resphera Biosciences, Baltimore, MD USA; 2 Department of Internal Medicine/Infectious Diseases Division, University of Michigan Medical School, Ann Arbor, MI USA; 3 Baylor University, Waco, TX USA

## Abstract

Microbiome profiling through 16S rRNA gene sequence analysis has proven to be a useful research tool in the study of *C. difficile* infection (CDI); however, CDI microbiome studies typically report results at the genus level or higher, thus precluding identification of this pathogen relative to other members of the gut microbiota. Accurate identification of *C. difficile* relative to the overall gut microbiome may be useful in assessments of colonization in research studies or as a prognostic indicator for patients with CDI. To investigate the burden of *C. difficile* at the species level relative to the overall gut microbiome, we applied a high-resolution method for 16S rRNA sequence assignment to previously published gut microbiome studies of CDI and other patient populations. We identified *C. difficile* in 131 of 156 index cases of CDI (average abundance 1.78%), and 18 of 211 healthy controls (average abundance 0.008%). We further detected substantial levels of *C. difficile* in a subset of infants that persisted over the first two to 12 months of life. Correlation analysis of *C. difficile* burden compared to other detected species demonstrated consistent negative associations with *C. scindens* and multiple *Blautia* species. These analyses contribute insight into the relative burden of *C. difficile* in the gut microbiome for multiple patient populations, and indicate that high-resolution 16S rRNA gene sequence analysis may prove useful in the development and evaluation of new therapies for CDI.

## C. difficile: Investigating infection in the gut

A detailed analysis of the prevalence of the bacterium *Clostridium difficile* in the gut will aid research and could improve clinical care. *C. difficile* infection is a major global healthcare problem, causing 29,000 deaths each year in the USA alone. It is frequently acquired by hospitalized patients, especially after antibiotic treatment. Researchers in the USA, led by James White at Resphera Biosciences in Baltimore, Maryland, used high-resolution 16S rRNA sequence analysis of gut microbial communities to characterize *C. difficile* levels in several patient populations. The results gave insights into the burden of *C. difficile* during infection relative to healthy control populations. They also revealed substantial levels of *C. difficile* in some infants through the first year of life. The authors suggest the analysis technique they used may help guide the development of new treatments for these difficult infections.

## Introduction

*Clostridium difficile* infection (CDI) poses a major healthcare burden to the global population, with an estimated 450,000 cases and 29,000 deaths in the United States annually.^1,2^ CDI is often associated with antibiotic treatment and is frequently acquired by patients during hospitalization. Multiple diagnostic tests for CDI are available and hospitals commonly use a combination of enzyme immunoassay (EIA) and glutamate dehydrogenase (GDH) testing in tandem with real-time polymerase chain reaction (PCR) for increased sensitivity and shorter turnaround time.^3^

After diagnosis, patients with CDI are typically treated with metronidazole and/or vancomycin depending on symptom severity.^3^ Treatment failure is estimated to occur in 20% of patients, resulting in a recurrent CDI population that may require other treatment strategies.^4,5^ The development of microbial-based therapeutics, such as fecal microbiota transplantation (FMT) and combinations of selected microbes for the treatment of recurrent CDI suggests that mixtures of commensal microbes may be routinely utilized in the future as an alternative to powerful antibiotics.^6,7^

Microbiome profiling through 16S rRNA gene sequencing has proven to be a valuable tool to characterize the diversity and composition of gut microbial communities, including in studies of CDI development and recurrence.^8^ Given the intricate relationship between the gut microbiota and CDI, accurate identification of *C. difficile* directly from 16S rRNA profiles in patient populations could be a valuable measure in future studies. However, a fundamental challenge to studying *C. difficile* through these approaches has been the level of taxonomic resolution provided through short 16S rRNA sequences. As a result, most microbiome sequencing studies of CDI utilize higher aggregate taxonomic categories (e.g., the *Clostridium* XI cluster, which encompasses many other organisms related to *C. difficile*) as a proxy for the organism itself or simply avoid quantification altogether.^9–17^

Here we utilize a high-resolution method (Resphera Insight) for assigning species-level context to 16S rRNA gene sequence data to estimate *C. difficile* burden in different patient populations. This method was recently validated for detection of *Listeria monocytogenes* ^18^ and *Salmonella enterica*,^19,20^ and was applied in this study to determine the relative abundance of *C. difficile* in several clinically relevant patient groups. Re-examining published 16S rRNA gene sequence datasets has confirmed previous associations of *C. difficile* with *C. scindens*, and identified new positive and negative correlations of *C. difficile* with other species, both of which may help provide insight into community aspects of *C. difficile* colonization and resistance against CDI.

## Results

### Evaluation of sensitivity and specificity for *C. difficile* identification

One of the challenges of 16S rRNA gene sequencing is the limited information available in these short DNA fragments to distinguish related microbial members below the genus-level. To accurately assess *C. difficile* at the species level from 16S rRNA gene sequence data, we used a method developed specifically for species level characterization (Resphera Insight, see Methods). We first validated this approach by obtaining full-length 16S rRNA gene sequences from 804 novel *C. difficile* isolates derived from multiple sources, and subsequently simulated noisy 16S rRNA gene sequence reads for taxonomic assignment (see Methods). Performance was measured using the Diagnostic True Positive Rate (DTP), defined as the percentage of sequences with an unambiguous assignment to *C. difficile*. The method achieved an average DTP of 99.9% (ranging from 98.92 to 100% per isolate, Table S1), indicating sufficient sensitivity to detect *C. difficile* from short 16S rRNA gene sequence reads.

In addition to establishing sufficient sensitivity to detect *C. difficile*, we also sought to evaluate false positive rates in which the method incorrectly assigns a sequence to *C. difficile*. As this species is a member of the *Clostridium* XI cluster, a false positive assessment was performed based on in silico simulations of 22 other members of this group, including the very similar *Clostridium irregulare*. Simulating 10,000 16S rRNA gene sequence reads per species with a 0.5% error rate, 20 of 22 species resulted in zero false positive assignments to *C. difficile*, with the highest false positive rate (0.07%) attributed to *Clostridium irregulare* (Table 1).

**Table 1:** False positive rates for 22 related species

Species from clostridium XI cluster	False positive rate (%)
*Clostridium acidurici*	0
*Clostridium bartlettii*	0.01
*Clostridium bifermentans*	0
*Clostridium caminithermale*	0
*Clostridium ghoni*	0
*Clostridium glycolicum*	0
*Clostridium halophilum*	0
*Clostridium hiranonis*	0
*Clostridium irregulare*	0.07
*Clostridium litorale*	0
*Clostridium mangenotii*	0
*Clostridium maritimum*	0
*Clostridium mayombei*	0
*Clostridium metallolevans*	0
*Clostridium paradoxum*	0
*Clostridium purinilyticum*	0
*Clostridium rectum*	0
*Clostridium ruminantium*	0
*Clostridium sordellii*	0
*Clostridium thermoalcaliphilum*	0
*Clostridium ultunense*	0
*Clostridium venationis*	0

### Representation of *C. difficile* relative to the microbiota in adult cases of CDI and healthy individuals

To examine the presence of *C. difficile* in different human populations, we re-examined existing published 16S rRNA gene sequencing datasets with our validated method. We first compared the relative abundance of *C. difficile* across a cohort of healthy individuals to two cohorts of patients diagnosed with CDI (symptomatic index cases) from Seekatz et al.^10^ and Khanna et al.^21^ (Table S2). The Seekatz protocol for CDI diagnosis followed a two-stage algorithm employing enzyme immunoassay for GDH antigen and toxins A and B, with confirmation of tcdB gene presence via PCR if toxin and GDH results were discordant; the Khanna et al. protocol for CDI diagnosis was not reported in the original publication. The healthy patient cohort and Seekatz CDI datasets were generated using equivalent processing and sequencing methods.^10^ Average analyzed sequencing depths per sample for CDI and healthy groups were 16,114 and 14,937, respectively.

**Fig. 1:**
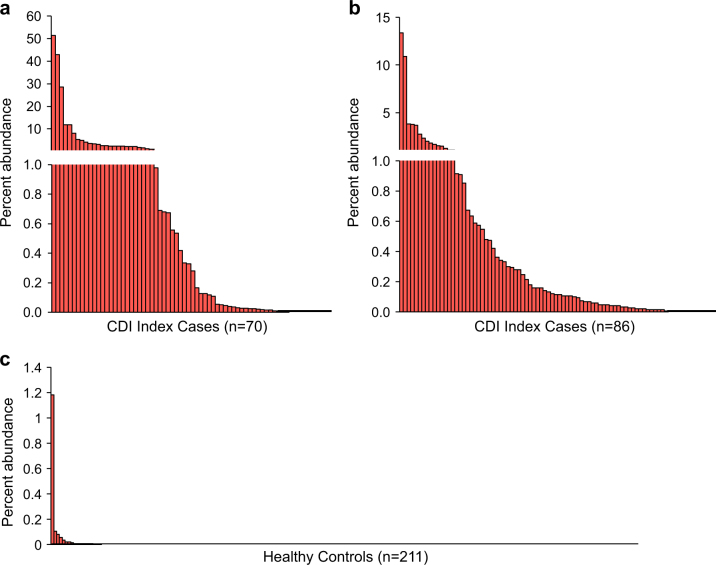
Relative burden of *C. difficile* in the gut microbiome of two cohorts of CDI index patients and healthy controls. **a** Index cases of recurrent CDI (Seekatz et al.) and **b** CDI index patients (Khanna et al.) frequently harbored moderate to high levels of *C. difficile*. **c** Healthy controls. Overall 91.5% of controls had no detectable *C. difficile* and 0.9% maintained *C. difficile* levels higher than 0.1%

Overall, *C. difficile* was detected in 58 of 70 CDI index patients (82.9%) in the Seekatz study with an average abundance of 3.04% (Fig. 1a). In the Khanna dataset, *C. difficile* was detected in 73 of 86 CDI index patients (84.9%) with an average abundance of 0.76% (Fig. 1b). Among healthy controls, only 18 of 211 (8.5%) harbored detectable levels of *C. difficile*, with an average abundance of 0.008%, significantly less than both Seekatz and Khanna index cases (*P* < 2e-16; Mann–Whitney test).

We were further interested in determining whether the ability to detect *C. difficile* or varying levels of *C. difficile* relative abundance from 16S rRNA gene sequences was related to disease outcome. The Seekatz dataset included samples collected from patients that went on to develop recurrent CDI, a serious outcome following primary diagnosis, or from patients who were later reinfected with CDI beyond the standard time recurrence window.^10^ Additionally, a severity score^22^ was available for some of the patients. Across the full Seekatz CDI positive sample set, our method detected *C. difficile* above 0.1% abundance in 59.2% of samples (Table 2). On average, patients with CDI for index (at primary diagnosis), recurrence or reinfection events had *C. difficile* abundances greater than 1% regardless of the calculated severity status using Infectious Diseases Society of America (IDSA) standards. We found no significant associations of *C. difficile* abundance with IDSA severity status among index samples or at the time of recurrence or reinfection (*P* > 0.05, Mann–Whitney test).

**Table 2:** *C. difficile* relative abundances in cases of CDI from Seekatz et al.^10^ compared to healthy controls

		Detected *C. difficile* levels (% abundance)			Distribution (% of patients)				
	IDSA category	Mean	Median	Max	*N*	CD > 0%	CD > 0.01%	CD > 0.05%	CD > 0.10%
Index case	Non-severe	2.3	0.33	42.9	38	81.6%	78.9%	65.8%	63.2%
	Severe	4.07	0.09	51.5	30	86.7%	76.7%	53.3%	50.0%
	NA	1.61	1.61	3.2	2	50.0%	50.0%	50.0%	50.0%
Recurrence	Non-severe	9.36	3.84	42.6	20	85.0%	80.0%	75.0%	75.0%
	Severe	1.19	0.64	7.1	9	77.8%	66.7%	66.7%	66.7%
	NA	1.53	0.07	11.3	9	55.6%	55.6%	55.6%	44.4%
Reinfection	Non-severe	3.38	0.55	19.3	16	62.5%	62.5%	56.3%	56.3%
	Severe	3.29	0.54	14.5	14	85.7%	71.4%	64.3%	57.1%
	NA	2.8	0.21	10.1	9	77.8%	77.8%	77.8%	55.6%
Healthy controls	No disease	0.008	0	1.2	211	8.5%	3.8%	1.9%	0.9%

*NA* data not available, *CD C. difficile* abundance

### Representation of *C. difficile* relative to the microbiota in infants

To assess the levels of *C. difficile* carriage among infants relative to the total gut microbiome, we re-examined 16S rRNA gene sequence datasets describing longitudinal studies of pre-term infants in the neonatal intensive care unit (NICU) by Zhou et al.^23^ and a single infant profiled during the first 18 months of life by Davis et al.^16^ In the Zhao dataset, 12 necrotizing enterocolitis (NEC) cases and 26 age-matched controls (all treated at Brigham and Women’s Hospital NICU, Boston, MA) were sequenced with an average of seven samples per subject. The Davis asymptomatic case study consisted of profiling 50 fecal samples over time, during which researchers noted colonization switching between toxigenic and non-toxigenic strains and observed 100,000-fold fluctuations of *C. difficile* spore counts.^16^

**Fig. 2:**
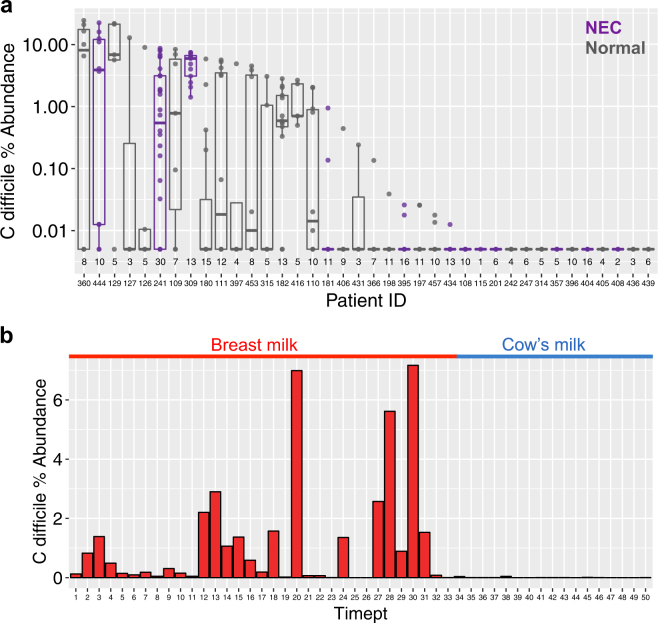
Distribution of *C. difficile* during longitudinal gut microbiome sampling of infants. **a** Pre-term infants in a NICU, including those developing necrotizing enterocolitis (purple) and normal (grey). Each boxplot reflects a single patient with multiple time points (total samples per patient shown along the *x*-axis). **b** A longitudinal case study of an infant before (red) and after (blue) weaning during the first 18 months of life. During the transition to cow’s milk, *C. difficile* relative abundance fell to undetectable levels

In these two 16S rRNA gene sequence datasets, moderate levels of *C. difficile* (>1.0% abundance) appeared consistently within infants over time. In the Zhao dataset, *C. difficile* was detected in 25 of 38 (66%) infants, including 6 of 12 (50%) infants with NEC, and 19 of 26 (73%) normal infants. There was no significant difference in overall *C. difficile* presence between NEC and normal infants (*P* = 0.27, Fisher’s exact test), and both groups maintained statistically similar *C. difficile* abundance distributions relative to their total gut microbial communities under multivariate regression after adjustment for patient source (Fig. 2a). As the original Davis case study determined *C. difficile* carriage using spore counts and GDH concentration, we detected substantial representation of *C. difficile* (up to 7.1% abundance) until the time of weaning and transition to cow’s milk (Fig. 2b). We further found a statistically significant correlation between our *C. difficile* relative abundance estimates and GDH concentration measurements from the Davis study (Spearman correlation = 0.817; *P* = 5e-13).

### Correlations of *C. difficile* with other bacterial species

Recent studies in animal models have indicated that certain species may generate metabolites that inhibit *C. difficile*, such as the production of secondary bile acids by *C. scindens*.^15^ However, previous studies correlating the abundance of *C. difficile* with other taxa did not utilize the microbiome-based abundances directly, but rather quantified *C. difficile* abundance through other means such as real-time PCR, colony forming units through culture, measuring GDH concentration or spore counts.^15–17^

**Fig. 3:**
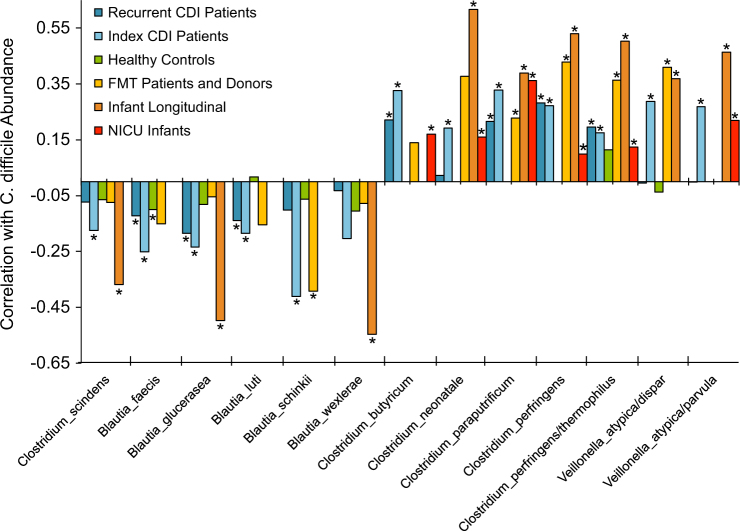
Correlation analysis identifies species positively or negatively associated with *C. difficile*. The CCREPE N-dimensional checkerboard score (*y*-axis) incorporates the ratio of co-variation to co-exclusion patterns normalized to a range of (−1, +1). In addition to *C. scindens*, we identify significant negative correlations with *C. difficile* for members of *Blautia* and positive correlations with other *Clostridia* and *Veillonella spp*. (*P ≤ 0.05). Ambiguous species level assignments are denoted by slashes. Key for re-analyzed datasets from the following studies: Recurrent CDI=^10^, Index CDI=^21^, FMT=^9^, Infant longitudinal=^16^, NICU=^23^ (Table S2)

We sought to determine whether high-resolution analysis of the 16S rRNA gene sequence data itself could reveal the same associations, and perhaps other relevant species. Computing correlations using Compositionality Corrected by REnormalization and PErmutation (CCREPE)^24^ across our re-analyzed cohorts, we found a significant negative association between *C. difficile* and *C. scindens* for the Khanna CDI patient cohort and the Davis infant longitudinal study (*P* < 0.02 for both datasets), with a supporting trend in the other studies (Fig. 3, Table S3). Additionally, multiple members of *Blautia spp*. displayed a consistent negative correlation like that of *C. scindens* (Fig. 3, Table S3). In contrast, other *Clostridia* such as *C. neonatale* and *C. paraputrificum* and members of *Veillonella* showed strong positive associations with *C. difficile* abundance. In silico simulations of noisy 16S rRNA gene sequence reads from these species confirmed a low mis-assignment rate (average 0.08%; see Table S4).

## Discussion

In this study, we sought to identify species-level abundances of *C. difficile* in 16S rRNA gene sequence datasets from different patient populations using a validated algorithm (Resphera Insight). Similar to previous studies of *Listeria monocytogenes* ^18^ and *Salmonella enterica*,^19,20^ validation using a high-resolution taxonomic assignment method from 804 novel *C. difficile* isolates established an overall sensitivity of 99.9% with a marginal false positive rate less than 0.1%, suggesting that *C. difficile* could be distinguished from other related microbiota members.

Compared to the microbiota of healthy individuals, we observed a higher presence and relative abundance of *C. difficile* in microbiota data collected from two CDI patient cohorts. 8.5% of healthy individuals were positive for *C. difficile* using our approach, supporting previous epidemiological assessments of asymptomatic carriage rates.^25–28^ Although analysis of CDI datasets revealed a wide distribution of *C. difficile* relative abundances (ranging from virtually undetectable to above 50% of total sequences), the relative abundance of detected *C. difficile* in relation to other members of the microbiota was significantly lower in healthy individuals than that of CDI patients. The ability to assess *C. difficile* levels as part of the microbiota community is potentially more important within population surveys compared to diagnosis using traditional PCR or GDH/EIA tests that merely account for the presence of *C. difficile* using toxin B or GDH as a proxy.

While detection of *C. difficile* from 16S rRNA gene sequence data is limited by sequencing depth, our results suggest that *C. difficile* does not generally reside in healthy adults. In contrast, we did not detect *C. difficile* in all patients with CDI. The relative presence of *C. difficile* in these patients is likely below the detection limit given the available sequencing depth, however some of the samples collected from patients in the Seekatz dataset were collected during antibiotic treatment, thus potentially limiting growth of *C. difficile* during those time points. Indeed, Seekatz et al. report that they were unable to retrieve *C. difficile* strains from all patient time points via anaerobic cultivation, generally the gold standard for *C. difficile* detection and diagnosis.

In a third cohort of 14 recurrent CDI patients receiving fecal microbiota transplantation from nine healthy donors (FMT; Table S2, Fig. 3), *C. difficile* was less frequently detected than the Seekatz and Khanna index CDI patient groups. Only 4 of 14 FMT patients had any detectable levels of *C. difficile* before treatment, and 3 of 14 had observations of *C. difficile* post-FMT. Notably, Resphera Insight detected *C. difficile* presence in both patients who went on to develop symptomatic CDI post-FMT (recipient IDs 005 and 006).^9^ Prior to FMT, all patients were treated with vancomycin (125 mg 4× per day) for at least 4 days before and the day of transplantation. Thus, we attribute the reduced detection of *C. difficile* in this cohort to differences in patient treatment before sampling.

Applying our approach to a longitudinal dataset of 38 premature infants in a single NICU, we identified *C. difficile* in two-thirds of this patient cohort. Asymptomatic carriage of *C. difficile* among infants has been observed to be higher than for adults, and it remains unknown whether infant cases of CDI represent true disease.^29,30^ While CDI testing of infants is not recommended,^30^ recent epidemiological studies indicate 26% of children hospitalized with CDI are infants under 12 months of age, and 5% are neonates.^31^ In one study of 753 pediatric patients 0 to 12 years of age, 2.9% of CDI outpatients, 4.6% of CDI inpatients, and 6.6% of healthy controls were positive for *C. difficile* toxin B.^32^ Another recent study of *C. difficile* in 338 healthy infants (<2 yrs) in the United Kingdom found 10% were colonized at enrollment with a toxigenic strain, and 49% became colonized with a toxigenic strain post-enrollment.^33^ Symptomatic *Clostridium difficile* infections are believed not to occur in infants due to the expected lack of specific toxin receptors and under-developed signaling pathways in the gut; however, these proposed mechanisms have not been rigorously evaluated in studies of humans.^34–36^ Multiple case studies have argued that CDI can occur in this patient population,^36^ and there is ongoing debate about the appropriate policy for treatment of symptomatic children who test positive for *C. difficile*.^37,38^

Our analysis of an infant case study of asymptomatic colonization during the first 18 months of life identified a reduction in *C. difficile* relative abundance after abrupt transition from human milk to cow’s milk. Yet in a large longitudinal study by Stoesser and colleagues, multivariate analysis demonstrated that breastfeeding (mixed with formula or exclusively) was protective against asymptomatic *C. difficile* colonization.^33^ As noted by Davis and colleagues,^16^ *C. difficile* does not carry the functional capacity for cleaving monosaccharides from oligosaccharide side chains and thus depends on the generation of monomeric glucose by other commensal members of the gut microbiome.^39^ Additionally, *C. difficile* relies on sialic acid as a carbon source for expansion made available by other commensals such as *Bifidobacterium* species.^40^ Therefore, the reduction of *C. difficile* after transition to cow’s milk is potentially the result not of milk source alone, but shifting microbial community composition and the presence of substrates by which *C. difficile* may thrive.

We were also able to identify a significant negative correlation between the abundance of *C. difficile* and *C. scindens* in one of the CDI cohorts, confirming similar trends reported by Buffie et al.^15^ *C. scindens*, a secondary bile acid producer of deoxycholic acid which has been shown to protect against CDI, may have important translational implications.^13,41^ New and consistent negative correlations were also identified between *C. difficile* and multiple species within the *Blautia* genus including *B. faecis*, *B. luti*, *B. schinkii*, and *B. wexlerae*. Notably, some members of the *Blautia* genus are known for 7α-dehydroxylating activity of primary bile acids,^42–44^ however this remains to be evaluated for the species we identified in this study. These data suggest that species other than *C. scindens* may provide relevant functional capabilities in the context of CDI and prove to be informative in the development of future microbial-based therapeutics. One exception to these findings was the lack of negative correlations identified within the NICU infant cohort, which can be attributed to the very limited observations of these *Blautia* species and *C. scindens* in the overall dataset (Table S3). Indeed, among the 322 NICU infant samples analyzed, only *B. luti* and *B. wexlerae* were observed at all, and only in 5 (1.6%) and 2 (0.6%) samples, respectively, which precluded their evaluation with the CCREPE method.

While microbiome profiling through 16S rRNA gene sequencing is unlikely to replace existing methods for routine diagnosis of CDI, sequence-based assessment of *C. difficile* levels in the context of microbiota profiling rather than presence alone may prove valuable in surveillance of *C. difficile* in patient populations, prediction of disease outcome, or the development of new therapies for CDI. Although our study is limited to 16S rRNA gene-based identification of *C. difficile* and cannot predict whether a strain produces toxin or carries a functional pathogenicity locus,^45^ consideration for accurate identification of *C. difficile* and related members may be useful in assessing clinical outcomes of new microbial therapies that rely on 16S rRNA gene sequencing to validate recovery of the microbiota.

## Methods

### Validation of Resphera Insight for identification of *C. difficile*

Whole-genome shotgun sequence datasets available from (i) The Wellcome Trust Sanger Institute and (ii) The University of Maryland Institute for Genome Sciences designated as novel *C. difficile* isolates were downloaded from the NCBI Sequence Read Archive (see Table S1 for accessions), trimmed for quality using Trimmomatic^46^ and assembled into contigs using Minia.^47^ Contigs containing portions of 16S rRNA genes were identified using BLASTN^48^ and extracted for amplicon simulations. For each isolate, we subsequently simulated 16S rRNA amplicon sequence reads (10,000 per isolate) from the V4 region (the primary amplicon region selected in the real datasets) with a random nucleotide error rate of 0.5%. The Diagnostic True Positive Rate was computed as the percentage of sequences unambiguously assigned by Resphera Insight to *C. difficile*.

For false positive assessment, simulated V4 sequences were generated from reference 16S rRNA genes for 22 unique species within the *Clostridium* XI cluster (10,000 per species, 0.5% nucleotide error rate). False positives were defined as unambiguous assignments to *C. difficile*.

### Processing of 16S rRNA gene sequence datasets

Raw 16S rRNA gene sequence datasets were processed as follows: Raw overlapping paired-end reads were merged into consensus fragments by FLASH^49^ requiring a minimum 20 bp overlap with 5% maximum mismatch density, and subsequently filtered for quality (targeting error rates < 1%) and length (minimum 200 bp) using Trimmomatic^46^ and QIIME.^50^ Spurious hits to the PhiX control genome were identified using BLASTN and removed. Sequences were then trimmed of their associated primers, evaluated for chimeras with UCLUST (de novo mode),^51^ and screened for human-associated contaminants using Bowtie2^52^ searches of NCBI *Homo sapiens* Annotation Release 106. Mitochondrial contaminants were detected and filtered using the RDP classifier^53^ with a confidence threshold of 50%, and passing high-quality 16S rRNA gene sequences were subsequently assigned to a high-resolution taxonomic lineage using Resphera Insight (Baltimore, MD).^18–20,54,55^ Briefly, the method relies on (i) a manually curated 16S rRNA gene database including 11,000 unique species and (ii) a hybrid global-local alignment strategy to assign sequences a species-level taxonomic lineage. While the method attempts to achieve species-level resolution, if the internal statistical model indicates uncertainty in final species membership, the tool minimizes false positives by providing “ambiguous assignments” i.e., a list of species reflecting all relevant candidates. For example, if a 16S rRNA gene fragment is ambiguous between *Veillonella atypica* and *Veillonella dispar*, the algorithm will provide the ambiguous assignment: “*Veillonella_atypica:Veillonella_dispar*.”

### Statistical analyses

Correlations between *C. difficile* and other species were computed using CCREPE (v.1.10.0)^24^ (http://huttenhower.sph.harvard.edu/ccrepe). CCREPE (Compositionality Corrected by REnormalization and PErmutation) utilizes an N-dimensional extension of the checkerboard score particularly suited to similarity score calculations between compositions derived from ecological relative abundance measurements of co-occurrence or co-exclusion. Two sample statistical comparisons utilized the Mann-Whitney *U* test unless otherwise noted.

#### In silico evaluation for species identified in CCREPE analysis

For single species reported in CCREPE correlation analysis, we simulated noisy 16S rRNA gene sequences (V4 region; 0.5% error rate; 1000 seqs per species), and calculated the frequency of (1) assignments that included the correct species (allowing for ambiguous assignments), (2) unambiguous assignments to the correct species, and (iii) mis-assignments that did not include the correct species (Table S4).

### Ethics approvals and consent to participate

IRB approval and patient consent statements from each study: Recurrent CDI (Seekatz et al.^10^)—All subjects signed written consent to participate in this study. This study was approved by the University of Michigan Institutional Review Board (Study HUM33286; originally approved 8/26/2009).

Index CDI (Khanna et al.^21^)—We prospectively recruited 88 patients (median age 52.7 years, interquartile range 36.9–65.1; 60.2% female) with their first CDI episode (from 3/2012–9/2013) as identified from the Clinical Microbiology Laboratory at Mayo Clinic, Rochester, Minnesota and collected an aliquot from the stool samples that led to the diagnosis. Clinical data including demographics, hospitalization status, concomitant medications, CDI severity, laboratory parameters, prior and concomitant antibiotic use, initial CDI treatment, treatment response and recurrent CDI were obtained by a review of the electronic medical record.

NICU Infants (Zhou et al.^23^)—Samples were collected following a protocol that was approved by the Partner’ s Human Research Committee (IRB) for Brigham and Women’ s Hospital. All study procedures were approved by the IRBs at both Brigham and Women’ s Hospital in Boston, MA and at The Genome Institute in St. Louis, MO. The IRB deemed this study to be of minimal risk with no interaction and no intervention with human subjects and thus, was exempt from consent.

Infant Longitudinal (Davis et al.^16^)—The study was approved by the TechLab Institutional Review Board and included informed consent obtained from the mother.

FMT (Seekatz et al.^9^)—Informed consent was received from all participants under an approved Institutional Review Board (IRB) protocol at Essentia Health Duluth Clinic (IRB no. SMDC-09068; principal investigator, Timothy Rubin, FDA Investigational New Drug [IND] no. 15460).

Healthy Controls (Seekatz et al. submitted)—All subjects signed written consent to participate in this study. This study was approved by the University of Michigan Institutional Review Board (Study HUM33286; originally approved 8/26/2009).

### Data availability

NCBI BioProject accessions of publicly available 16S rRNA gene sequence datasets used in this study: PRJNA307992, PRJNA342347, PRJNA264177, PRJNA331150, PRJNA238042, and PRJNA386260 (Table S2).

## Electronic supplementary material

Supplementary Table 1

Supplementary Table 2

Supplementary Table 3

Supplementary Table 4
