# Supplementary material for: High-resolution profiling of the gut microbiome reveals the extent of Clostridium difficile burden
Source: NPJ Biofilms Microbiomes. 2017 Dec 5;3:35. doi: 10.1038/s41522-017-0043-0 (PMC5717231; doi:10.1038/s41522-017-0043-0)
Supplement: Supplementary file 2 — Supplementary Table 2 [file 41522_2017_43_MOESM2_ESM.pdf]

**Table S2. Fecal microbiome 16S rRNA amplicon sequence datasets analyzed in this study.**

| Study Name | Citation | Population / Disease Status (n=total samples analyzed for *C. difficile* in this study) | Primer Design | NCBI SRA Accession |
|---|---|---|---|---|
| Recurrent CDI | Seekatz, Anna Maria, et al. "Dynamics of the fecal microbiome in patients with recurrent and nonrecurrent Clostridium difficile infection." Genome medicine 8.1 (2016): 47. | Patients with recurrent CDI (n=147) | V4 | PRJNA307992 |
| Index CDI | Khanna, S., et al. "Gut microbiome predictors of treatment response and recurrence in primary Clostridium difficile infection." Alimentary pharmacology & therapeutics 44.7 (2016): 715-727. | Index cases of CDI (n=86) | V4 | PRJNA342347 |
| NICU Infants | Zhou, Yanjiao, et al. "Longitudinal analysis of the premature infant intestinal microbiome prior to necrotizing enterocolitis: a case-control study." PloS one 10.3 (2015): e0118632. | NICU patients sampled over time (n=322) | V3V5 | PRJNA264177 |
| Infant Longitudinal | Davis, Manli Y., et al. "Rapid change of fecal microbiome and disappearance of Clostridium difficile in a colonized infant after transition from breast milk to cow milk." Microbiome 4.1 (2016): 53. | Single infant over first 18 months of life (n=50) | V4 | PRJNA331150 |
| FMT Patients and Donors | Seekatz, Anna M., et al. "Recovery of the gut microbiome following fecal microbiota transplantation." MBio 5.3 (2014): e00893-14. | Fecal Microbiota Transplant donors and recipients over time (n=40) | V3V5 | PRJNA238042 |
| Healthy Controls | Seekatz, Anna M., et al. submitted | Healthy adult patients (n=211; prepared using same methodology as the Recurrent CDI study) | V4 | PRJNA386260 |
