# Supplementary material for: High-resolution profiling of the gut microbiome reveals the extent of Clostridium difficile burden
Source: NPJ Biofilms Microbiomes. 2017 Dec 5;3:35. doi: 10.1038/s41522-017-0043-0 (PMC5717231; doi:10.1038/s41522-017-0043-0)
Supplement: Supplementary file 3 — Supplementary Table 3 [file 41522_2017_43_MOESM3_ESM.pdf]

Table S3. Correlation analysis by CCREPE between C. difficile and other species across six cohorts. Taxonomic assignments separated by a colon denote ambiguous assignments at the species level. Pvalues are shown in the bottom sub-table. Cells with NA indicate that taxa were not considered in that cohort due to low frequency counts or complete absence of that taxon.

Correlations with C difficile and Pvalues	Species	Recurrent CDI Patients	Index CDI Patients	Healthy Controls	FMT Patients and Donors	Infant Longitudinal	NICU Infants
**CCREPE CORRELATION**	Clostridium_scindens	-0.072319774	-0.17524347	-0.06406048	-0.074627789	-0.367704526	NA
**P VALUES**	Clostridium_scindens	0.262738308	0.013608847	0.095087189	0.672633028	0.000528427	NA
**CCREPE CORRELATION**	Blautia_faecis	-0.12178931	-0.25149598	-0.100438548	-0.151436548	NA	NA
**P VALUES**	Blautia_faecis	0.049343366	0.000578401	0.053262603	0.243909771	NA	NA
**CCREPE CORRELATION**	Blautia_glucerasea	-0.184077352	-0.23483875	-0.082015183	-0.053841312	-0.496846517	NA
**P VALUES**	Blautia_glucerasea	0.039505473	0.000257132	0.137957996	0.763404016	2.83E-08	NA
**CCREPE CORRELATION**	Blautia_luti	-0.138850169	-0.18528676	0.017033438	-0.153383223	NA	NA
**P VALUES**	Blautia_luti	0.048662367	0.034084423	0.900390219	0.436539266	NA	NA
**CCREPE CORRELATION**	Blautia_schinkii	-0.101639129	-0.41011422	-0.062964985	-0.391659326	NA	NA
**P VALUES**	Blautia_schinkii	0.207025506	1.24E-07	0.763576227	0.001695299	NA	NA
**CCREPE CORRELATION**	Blautia_wexlerae	-0.031765497	-0.20380662	-0.1052549	-0.078266048	-0.546450077	NA
**P VALUES**	Blautia_wexlerae	0.987534153	0.075750259	0.240999066	0.540246235	9.95E-10	NA
**CCREPE CORRELATION**	Clostridium_butyricum	0.220849423	0.32599151	NA	0.140028008	NA	0.170289
**P VALUES**	Clostridium_butyricum	0.001049585	0.000151065	NA	0.308405492	NA	0.0003545
**CCREPE CORRELATION**	Clostridium_neonatale	0.02195839	0.192689843	NA	0.377964473	0.616485947	0.1600567
**P VALUES**	Clostridium_neonatale	0.598326513	0.001766987	NA	0.082776566	3.43E-10	0.0034197
**CCREPE CORRELATION**	Clostridium_paraputrificum	0.216674454	0.32736945	NA	0.227377148	0.389719626	0.3616332
**P VALUES**	Clostridium_paraputrificum	5.73E-05	7.79E-05	NA	0.029201731	4.75E-05	4.40E-13
**CCREPE CORRELATION**	Clostridium_perfringens	0.282462897	0.272547168	NA	0.427668602	0.530704798	0.0991844
**P VALUES**	Clostridium_perfringens	4.90E-06	0.007319837	NA	0.011664925	1.57E-08	0.0384878
**CCREPE CORRELATION**	Clostridium_perfringens:Clostridium_thermophilus	0.195161644	0.175068625	0.11493353	0.363855531	0.503437907	0.1248443
**P VALUES**	Clostridium_perfringens:Clostridium_thermophilus	0.000540734	6.79E-05	0.103897318	0.019995972	2.13E-06	0.0079118
**CCREPE CORRELATION**	Veillonella_atypica:Veillonella_dispar	-0.004691019	0.286596927	-0.037189904	0.409852416	0.368224299	NA
**P VALUES**	Veillonella_atypica:Veillonella_dispar	0.65680325	0.016415127	0.662192729	0.002926516	2.67E-05	NA
**CCREPE CORRELATION**	Veillonella_atypica:Veillonella_parvula	-0.001045557	0.268197523	NA	NA	0.46435599	0.2194032
**P VALUES**	Veillonella_atypica:Veillonella_parvula	0.876727046	0.000480761	NA	NA	3.89E-05	0.0002418
**CCREPE CORRELATION**	Veillonella_denticariosi:Veillonella_parvula	-0.015168716	0.216374847	NA	NA	0.378362368	0.0902
**P VALUES**	Veillonella_denticariosi:Veillonella_parvula	0.577497094	0.016816816	NA	NA	0.008773263	0.0640465
**CCREPE CORRELATION**	Veillonella_dispar	-0.078992498	0.258182038	0.076311279	0.19626011	0.357009346	0.1859668
**P VALUES**	Veillonella_dispar	0.223255648	0.002447415	0.593644078	0.079622709	0.003182838	1.23E-06
