# Supplementary material for: High-resolution profiling of the gut microbiome reveals the extent of Clostridium difficile burden
Source: NPJ Biofilms Microbiomes. 2017 Dec 5;3:35. doi: 10.1038/s41522-017-0043-0 (PMC5717231; doi:10.1038/s41522-017-0043-0)
Supplement: Supplementary file 4 — Supplementary Table 4 [file 41522_2017_43_MOESM4_ESM.pdf]

Table S4. *In silico* simulated read results for significant single species identified in CCREPE analysis. For simulated 16S rRNA gene sequences (V4 region; 0.5% error rate), we recorded how frequently (1) a sequence assignment included the correct species (allowing for ambiguous assignments), (2) unambiguous assignments to the correct species, and (iii) mis-assignments that did not include the correct species. We observe low mis-assignment rates for all species, as well as high unambiguous assignment rates with the exception of Clostridium_perfringens which maintained higher ambiguous assignments with Clostridium_thermophilus (e.g. Clostridium_perfringens:Clostridium_thermophilus).

Species	Total Simulated Sequences	Assignment includes correct species (%)	Uambiguously assigned to the correct species (%)	Misassigned at the species level (%)
Blautia_faecis	1,000	100.0%	100.0%	0.0%
Blautia_glucerasea	1,000	100.0%	100.0%	0.0%
Blautia_luti	1,000	100.0%	100.0%	0.0%
Blautia_schinkii	1,000	99.8%	99.8%	0.2%
Blautia_wexlerae	1,000	99.7%	99.5%	0.3%
Clostridium_butyricum	1,000	99.9%	99.9%	0.1%
Clostridium_neonatale	1,000	100.0%	100.0%	0.0%
Clostridium_paraputrificum	1,000	99.8%	99.8%	0.2%
Clostridium_perfringens	1,000	100.0%	2.6%	0.0%
Clostridium_scindens	1,000	99.9%	99.9%	0.1%
Veillonella_dispar	1,000	100.0%	100.0%	0.0%
